# Evolving dengue serotype distribution with dominance of dengue virus- 3 in Bangalore: critical insights for vaccine efficacy and implementation

**DOI:** 10.1016/j.lansea.2024.100485

**Published:** 2024-09-14

**Authors:** Shruthi Uppoor, Tina Damodar, Lonika Lodha, Madhusudhan Huluvadi Nagarajaiah, Reeta S. Mani

**Affiliations:** a H Siddaiah Referral Hospital, Bruhat Bengaluru Mahanagara Palike, Bangalore, India; b Department of Neurovirology, National Institute of Mental Health & Neurosciences, Bangalore, India; c Vector Borne Disease Control Programme, Bruhat Bengaluru Mahanagara Palike, Bangalore, India

India's substantial dengue disease burden, with an estimated 12.9 million primary infections annually, presents a significant public health challenge.^1^ This extensive disease prevalence, coupled with the complex immunological dynamics of dengue virus serotypes, poses considerable obstacles to the development and implementation of effective dengue vaccines in India.^2^ Therefore, as the country prepares for phase 3 trials of quadrivalent dengue vaccines, understanding region-specific circulating dengue virus serotypes is crucial.^3^

Southern India reports the highest dengue seroprevalence, estimated at 77%.^1^ At present, Karnataka, a southern Indian state, is witnessing a surge in dengue cases, with 9880 cases reported from January to July, 2024 by the Government of Karnataka, compared to 2271 cases in the same period last year. Bangalore, the state capital, has been particularly affected by this spike with 8160 cases reported in these 7 months.^4^

To assess the current serotype distribution, we analyzed 1600 samples from acute febrile illness patients across all eight zones of urban Bangalore (East, South, West, Bommanahalli, Dasarahalli, Mahadevapura, Rajarajeshwari Nagar and Yelahanka). Samples were collected through health centers under the municipal corporation, Bruhat Bengaluru Mahanagara Palike (BBMP), between April and July 2024 and tested at the Department of Neurovirology, National Institute of Mental Health and Neurosciences (NIMHANS). Of these, 549 (34.3%) tested positive for dengue NS1 antigen and/or anti-dengue IgM by ELISA. Real-time PCR analyses of 507 (92.3%) dengue NS1 positive samples revealed 228 (45%) were positive for DENV serotypes, showing a striking predominance of DENV-3 (182, 79.8%), followed by DENV-1 (27, 11.8%), DENV-2 (19, 8.3%), and DENV-4 (3, 1.3%). Additionally, 3 (1.3%) samples showed DENV-2 and DENV-3 co-infection (Fig. 1).

**Fig. 1:**
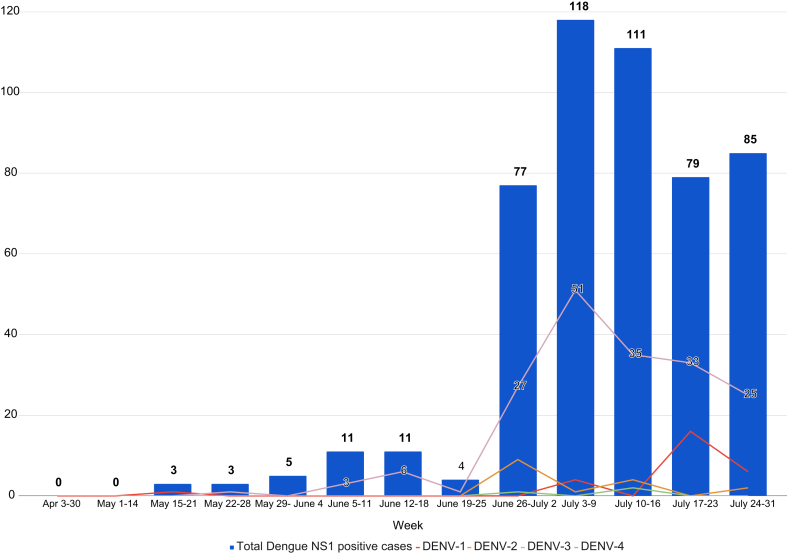
Dengue virus serotype positivity from April–July 2024, Bangalore (India). Figure shows week-wise positivity of DENV serotypes starting from May 15. There was no Dengue NS1 positivity in the month of April and first 2 weeks of May, which have been condensed in the figure.

Our findings may indicate a potential shift in serotype dominance towards DENV-3 from DENV-4, which has been the predominant serotype in Southern India since 2015, based on evolutionary studies of DENV.^5^ The dominance of DENV-3 is particularly significant given the challenges some vaccine candidates face in providing protection against this serotype. For instance, the TAK-003 (Takeda Pharmaceutical, Tokyo, Japan) vaccine showed limited efficacy of 62.6% against DENV-3 in trials conducted in dengue endemic regions of Asia and Latin America; while the Butantan–Dengue Vaccine (Instituto Butantan, Brazil and the National Institutes of Health (NIH) of the United States) trials in Brazil lacked efficacy data for both DENV-3 and DENV-4, as these serotypes were not in circulation during the follow-up period.^6^^,^^7^ The co-circulation of all four serotypes in this region reinforces the critical need for quadrivalent vaccines effective against all serotypes, especially considering India's significant contribution to global dengue burden.^1^ Moreover, the high prevalence of DENV-3 in this study presents a unique opportunity to generate robust efficacy data for this serotype in upcoming phase 3 trials in India, addressing a key gap identified in previous international trials.

To further enhance our understanding of dengue epidemiology and support vaccine development efforts, dengue virus sequencing is crucial. Sequencing data will allow for the correlation of disease severity and outcomes with circulating genotypes or mutations. Additionally, it may identify novel clades or genotypes and offer valuable insights into the molecular diversity, antigenic evolution, and vaccine escape mutants of dengue virus in this region.^5^^,^^8^ Recent challenges to the conventional belief of lifelong immunity against homologous dengue virus serotype infection underscore the need for a more detailed investigation of genotypes and clades.^9^ It has also been demonstrated, for dengue serotype 2, that genetic diversity within the serotype modulates antibody neutralization activity.^10^ By establishing a comprehensive genetic profile of circulating dengue viruses, we can better assess the potential effectiveness of current vaccine candidates and guide the development of future vaccines tailored to the specific strains prevalent in India.

## Contributors

Shruthi U: Conceptualization, Data curation, Formal analysis, Resources, Project administration, Writing—original draft, Writing—review & editing.

Tina Damodar: Conceptualization, Data curation, Formal analysis, Funding acquisition, Methodology, Project administration, Writing—original draft, Writing—review & editing.

Lonika Lodha: Data curation, Formal analysis, Methodology, Writing—original draft.

Madhusudhan H N: Resources.

Reeta Mani: Conceptualization, Funding acquisition, Supervision, Writing—review & editing.

## Declaration of interests

The authors declare no conflicts of interests related to this article, financial or otherwise.
